# Double DANTE‐PRESS: Development of a 1H‐MRS Multifrequency‐Selective Sequence for Simultaneous In Vivo Quantification of Glutathione and Glutamate at 7 Tesla

**DOI:** 10.1002/nbm.70385

**Published:** 2026-08-30

**Authors:** Kesavi Kanagasabai, Omer Oran, Lena Palaniyappan, Jean Théberge

**Affiliations:** ^1^ Imaging Program Lawson Research Institute London Ontario Canada; ^2^ Department of Medical Biophysics Western University London Ontario Canada; ^3^ Robarts Research Institute, Western University London Ontario Canada; ^4^ Siemens Medical Solutions USA, Inc. Palo Alto California USA; ^5^ Douglas Mental Health University Institute, Department of Psychiatry McGill University Montreal Quebec Canada; ^6^ Department of Medical Imaging St. Joseph's Health Care London Ontario Canada

**Keywords:** double DANTE‐PRESS, glutamate, glutathione, human brain, multifrequency‐selective pulse sequence, proton magnetic resonance spectroscopy

## Abstract

Glutamate and glutathione share a complex and tightly regulated cycle where imbalances can be a symptom of neurocognitive disorders. Magnetic resonance spectroscopy (1H‐MRS) is a noninvasive in vivo imaging technique used to quantify the concentration of human brain metabolites. Metabolite‐targeted techniques produce simple brain spectra that use simple fitting models. This reduces algorithmic variability and increases the precision of measurements of the metabolite(s) of interest. Common metabolite‐targeted techniques using spectral editing have drawbacks such as increased scan time and low signal‐to‐noise ratio efficiency and are prone to motion‐related subtraction artifacts. We introduce a custom single‐shot multifrequency‐selective sequence known as double‐DANTE‐PRESS that does not involve spectral editing. This sequence uses narrowband refocusing pulses allowing the users to refocus the signal from within two frequency passbands of interest while suppressing unwanted signals from outside the passbands. This produces simpler spectra mostly containing signals from metabolites of interest. In this study, we use double‐DANTE‐PRESS to acquire measurements of glutamate and glutathione simultaneously and report on its precision at 7 Tesla in phantoms and in vivo human brain. Double DANTE‐PRESS (TE/TR = 96 ms/3000 ms) was programmed within the software environment of the Siemens Magnetom 7 Tesla MR scanner at the Centre for Functional Metabolic Mapping of Western University (VE12U). Two scans were obtained on phantoms and in five healthy volunteers (20 × 20 × 20 mm^3^ voxel at the dorsal anterior cingulate cortex) as an in vivo proof‐of‐concept to refocus the glutamate multiplet at 2.35 ppm and glutathione singlet at 3.77 ppm simultaneously. In vivo, the mean inter‐individual %CV of glutathione and glutamate were less than 10% and 7%, respectively, and the mean %CRLBs of glutathione and glutamate were 10% and 5%. Double‐DANTE‐PRESS can allow for the simultaneous detection of glutamate and glutathione. This technique simplifies spectral quantification allowing for precise and simultaneous measurements. Future work includes a formal in vivo test–retest study and application to clinically driven research in patients with neurocognitive disorders.

## Graphical abstract

A single‐shot, multifrequency‐selective 1H‐MRS technique known as double Delays‐Alternating‐Nutation‐Tailored‐Excitation‐Point RESolved‐Spectroscopy (DANTE‐PRESS) can selectively refocus two frequencies of interest and simplify spectral modeling.

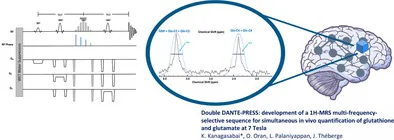

**Abbreviations**
1H‐MRS: proton magnetic resonance spectroscopy
Cr: phosphocreatine
CRLB: Cramer–Rao lower bound
CV: coefficient of variation
dACC: dorsal anterior cingulate cortex
DANTE‐PRESS: Delays Alternating with Nutations for Tailored Excitation Point RESolved Spectroscopy
FOV: field of view
FT: Fourier transform
FWHM: full‐width‐at‐half‐maximum
GABA: gamma‐amino butyric acid
Glu: glutamate
Gln: glutamine
GSH: glutathione
NA: number of averages
NAA: *N*‐acetylaspartate
NAAG: *N*‐acetyl‐aspartyl‐glutamate
PRESS: Point ReSolved Spectroscopy
pTx: parallel transmission
RF: radiofrequency
SNR: signal‐to‐noise ratio
SVS: single voxel spectroscopy
T1: longitudinal relaxation
T2: transverse relaxation
T: tesla
TE: echo time
TI: inversion time
TR: repetition time
TSP: 3‐(trimethylsilyl)‐propionic acid
VOI: volume of Interest
WET: water excitation transfer

## 1 Introduction

Glutamate (Glu), the most abundant excitatory neurotransmitter, is tightly regulated and/or replenished by a multitude of mechanisms such as the Glu‐Gln (Gln) shuttle, γ‐glutamyl cycle, and the glutathione (GSH) cycle [1, 2]. GSH is a tripeptide consisting of Glu, cysteine, and glycine where it often acts as an antioxidant that aids in drug detoxification while also hypothesized to serve as a physiologic reservoir of Glu in neurons [1, 2]. In neurocognitive and psychiatric disorders, excess of Glu leads to neuronal inflammation due to Glu excitotoxicity where GSH has therapeutic effects in counteracting the inflammation‐induced oxidative stress [3].

Single‐voxel proton magnetic resonance spectroscopy (1H‐MRS) is a noninvasive in vivo imaging technique used to quantify the concentration of human brain metabolites. Glu has five protons in an AMNPQ spin system resulting in three multiplets found in the brain spectrum: alpha at 3.74 ppm, gamma at 2.35 ppm, and beta at 2.12 ppm. GSH's cysteine moiety follows an ABX spin system where it contains a singlet at 3.77 ppm and three doublet‐of‐doublets at 2.93, 2.98, and 4.56 ppm [4]. GSH detection and quantification are often complicated with overlapping resonances from neighboring metabolites such as Glu, Gln, gamma‐amino butyric acid (GABA), and creatine. Currently, most MRS techniques targeting GSH involve either non‐frequency‐selective or metabolite‐selective techniques.

To achieve metabolite‐selectivity, MRS techniques often use spectral editing sequences where metabolite selectivity is achieved by utilizing its scalar (J) coupling network either through J‐difference‐editing or multiple‐quantum coherence filtering [5]. However, spectral editing techniques (1) increase scan time, (2) are more sensitive to motion artifacts, and (3) provide lower signal‐to‐noise ratio (SNR) efficiency. On the other hand, non‐frequency‐selective techniques are often optimized for one metabolite but reduce precision of other metabolites [6]. Furthermore, non‐frequency‐selective sequences produce complex spectra increasing the number of unknown parameters in the metabolite quantification model, which results in greater algorithmic variability. Point RESolved Spectroscopy (PRESS) is a non‐frequency‐selective technique whose timing parameters (echo time) are often optimized to target one metabolite of interest and produce complex spectra such that it would include over 20 metabolites to model along with their unique peak parameters (amplitudes, chemical shifts, linewidths, zero‐order phase, and first‐order phase).

Alternatively, using a multifrequency‐selective technique that is optimized to target two metabolites of interest simultaneously while suppressing unwanted signals can produce simpler spectra leading to a simpler spectral model with 2–4 metabolites to model along with their peak parameters. Assuming the number of acquired spectral points is the same, the model properly fits the data; the model with a reduced number of parameters will provide improved algorithmic variability (often measured via CRLBs) letting the study participants' variability be the dominant component of study statistical power. Therefore, there is an unmet need to develop a single‐shot multifrequency‐selective technique that can produce simple spectra and can be modelled using simpler fitting templates. In this study, we are targeting the GSH singlet at 3.77 ppm, which is often difficult to isolate due to overlapping resonances in the region.

Recently, we demonstrated the feasibility and reproducibility of Delays Alternating with Nutations for Tailored Excitation Point RESolved Spectroscopy (DANTE‐PRESS) on Glu and NAA in vivo at 7 Tesla (T) [7]. Building on this framework, this study is an evolution of single DANTE‐PRESS toward a simultaneous multifrequency‐selective sequence known as double DANTE‐PRESS. This sequence will only preserve the signal of two frequencies of interest while suppressing unwanted signals through a narrow‐band frequency‐selective refocusing pulse without using spectral editing, which relies on spin coherences evolving under J‐coupling [9, 10]. In addition to its multifrequency‐selectivity, double DANTE‐PRESS has several unique features that make it advantageous at 7 T. The DANTE pulse is a train of short hard pulses, which exhibit lower specific absorption rates (SAR) than a continuous Gaussian pulse. The DANTE pulse is an online generated pulse where the user can customize its features and perform fine‐tune calibrations in real‐time at the scanner.

### 1.1 Theory

**FIGURE 1:**
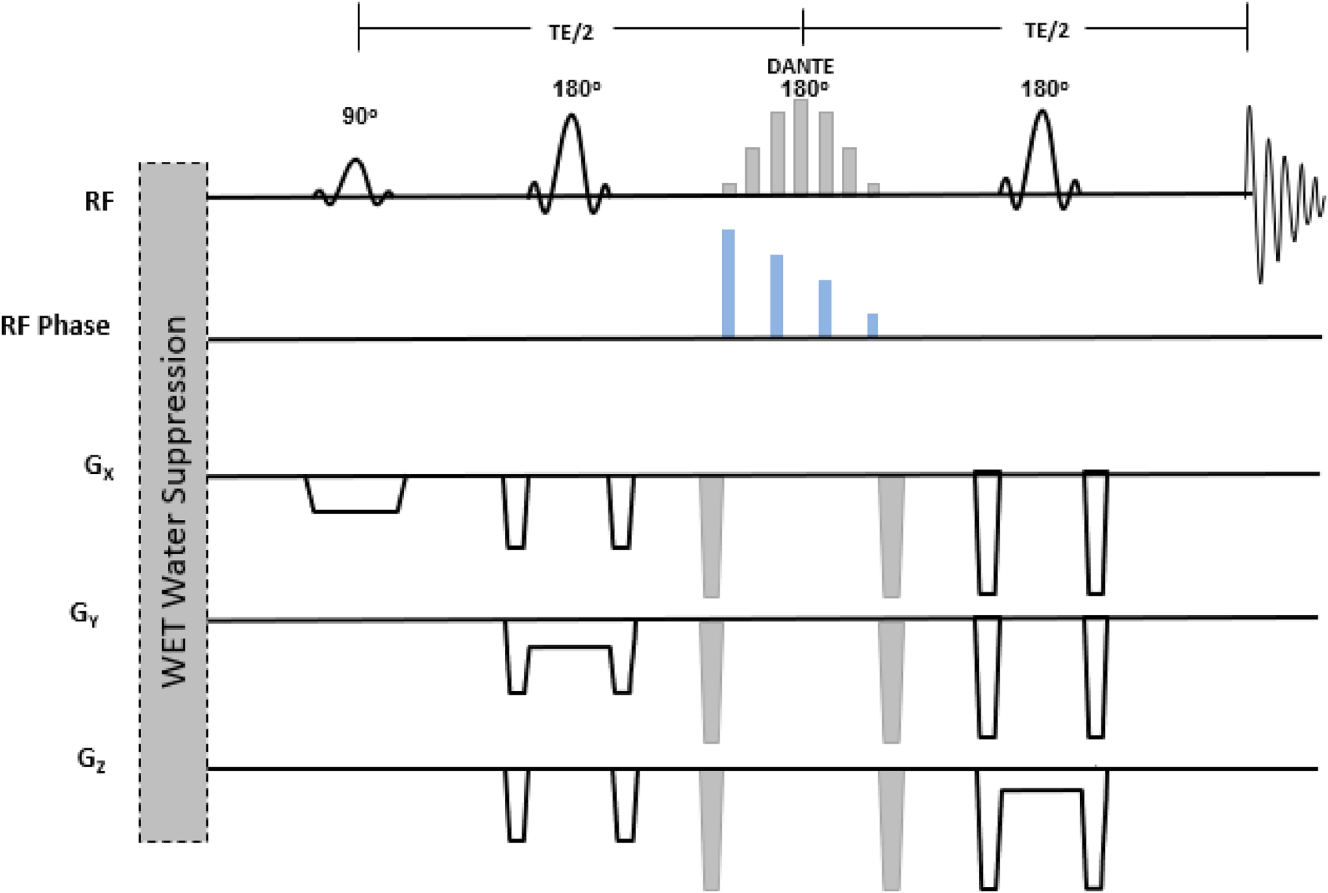
Double Delays Alternating with Nutations for Tailored Excitation Point RESolved Spectroscopy (DANTE‐PRESS) pulse sequence diagram. The sequence uses a 90°, slice‐selective Hanning‐filtered sinc pulse, followed by a slice‐selective 180° Mao refocusing pulse at TE/4, and a non‐slice‐selective 180° DANTE pulse with a nonzero linearly evolving phase at TE/2, and finally followed by a second 180° Mao refocusing pulse at 3TE/4. All the 180° pulses, including the DANTE pulse, are flanked by crusher gradients for coherence selection. Only spins within the narrow bandwidth of the DANTE pulse will be refocused.

In our previous single DANTE‐PRESS work, we described the core features of the DANTE pulse, a train of square pulses with a constant delay between the individual square pulses (Δ*t*) [7]. The same principles apply to double DANTE‐PRESS; however, a secondary off resonance excitation is achieved by applying a nonzero linear phase term to every other square pulse (Equation 1) where ∆ƒ corresponds to the frequency difference between the primary and secondary metabolite—an interleaved constant phase and linearly increasing phase. According to the Fourier theory, a phase term in one domain results in a shift in the other domain. Therefore, in a double DANTE pulse, you have a pulse train with a constant zero phase interleaved with a nonzero linearly evolving phase to achieve two frequency‐selective low‐power narrow excitation passbands (Figure 1).

$$∆f_{n}=∆Փ/\left(2\;\pi ∆t_{n}\right)$$

Equation (1): Analytical description of a secondary frequency offset produced by using a linearly evolving phase to every other short square pulse.

**FIGURE 2:**
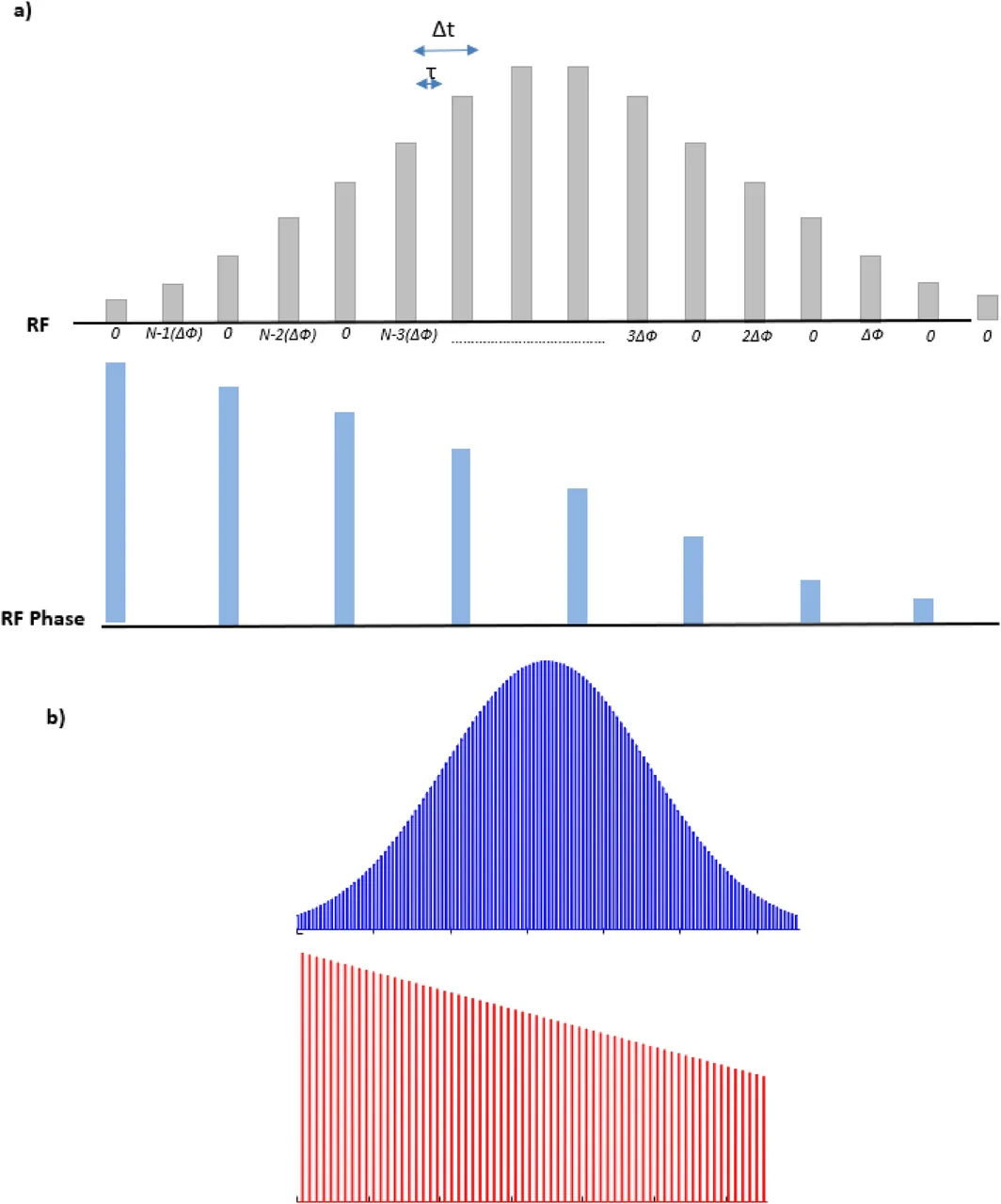
(a) Expanded view of the DANTE pulse waveform composed of multiple successive square pulses where *τ* is the square pulse duration (24 ms), ∆ *t* (165 μs) is the time between square pulses, and RF phase represents the nonzero linearly evolving phase applied to every other square pulse in the DANTE train to achieve the secondary passband frequency offset. (b) Expanded view of a MATLAB scaled simulation of the double DANTE pulse.

**FIGURE 3:**
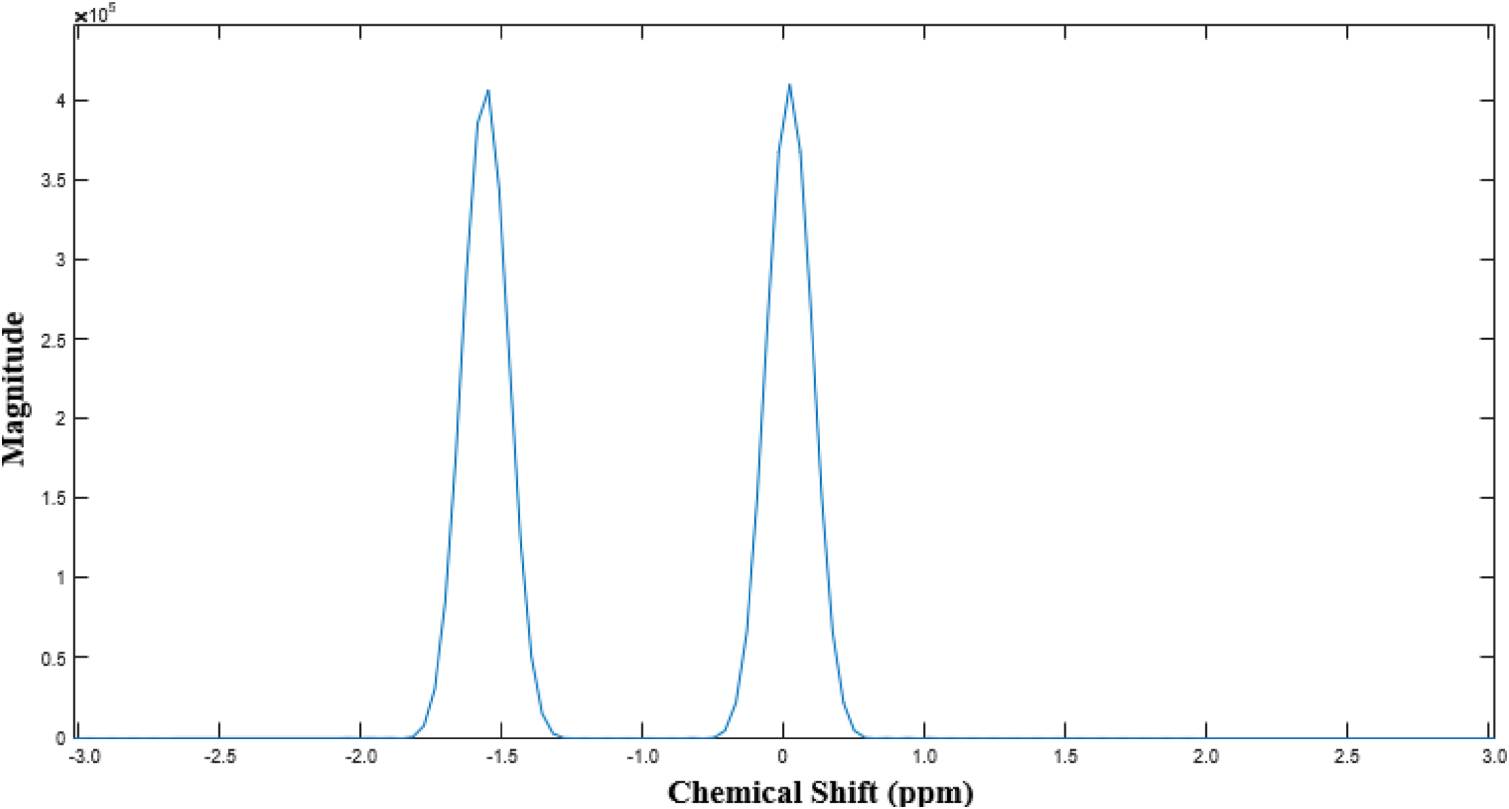
The frequency profile of the double DANTE pulse computed via the Fourier transform of the pulse train in Figure 1. The DANTE pulse duration is approximately 24 ms; the individual square pulses are 41.05 μs long and repeat every 164.2 μs. The amplitude of each square pulse is modulated by a sinc function with 14.0 kHz FWHM. It has a Gaussian amplitude modulation with truncation of the wings at 5% of the maximum amplitude. This produces a frequency profile with two primary passbands 423 Hz apart (1.42 ppm) and a full‐width half maximum of 56 Hz with secondary passbands located 2000 Hz away (not shown here).

With a linearly evolving phase, the frequency profile of the double DANTE produces two frequency‐selective passbands with one corresponding to the centre frequency and the second being with a frequency offset relative to the centre frequency (Figures 2 and 3).

### 1.2 Objective and Hypothesis

The first objective of this study is to introduce a novel multifrequency‐selective approach known as double DANTE‐PRESS and optimize its acquisition parameters to refocus Glu at 2.35 ppm (^4^C multiplet) and GSH at 3.77 ppm (singlet) simultaneously in phantoms and in vivo. The second objective is to perform a proof‐of‐concept and report on the precision of Glu and GSH concentration estimates applying the double DANTE‐PRESS approach in brain‐mimicking phantoms and human brain in vivo at 7.0 T.

It is hypothesized that double DANTE‐PRESS will achieve spectral signatures of Glu and GSH‐weighted simultaneously with a mean quantification precision (percent inter‐trial CV) < 10% in phantoms and in vivo.

## 2 Methods

In this study, the scanner hardware and shimming procedures were adopted from our previous single‐DANTE‐PRESS work (Kanagasabai et al., 2026) [7]. MR examinations were performed on the Siemens Healthineers MAGNETOM 7 T Plus (Forchheim, Germany) head‐only scanner (Centre for Functional and Metabolic Mapping, Robarts Research Institute, London, Ontario) using an eight‐transmit‐channel, head radiofrequency coil and 32‐channel receive coil [10]. We prescribed a 2.0 × 2.0 × 2.0 cm^3^ voxel within spherical water‐solution phantoms and in vivo. The DANTE‐PRESS parent sequence uses the Siemens' VE12U product PRESS sequence. B_1_ shimming involved an image‐based approach for the head and voxel B_0_ shimming involved the FASTESTMAP. FASTESTMAP was run 2–3 times until we observed a water linewidth under 15 Hz.

The first author (KK) developed and compiled the DANTE‐PRESS sequence along with custom adjustment loops for fine tune calibration of the excitation, refocusing, and frequency‐selective pulses for the Siemens MAGNETOM 7 T Plus (software baseline VE12U‐AP01) scanner.

### 2.1 Phantoms

Glu‐only and GSH‐only phantoms were built to validate FID‐A generated spectral fitting templates for Glu and GSH using double DANTE‐PRESS with a TE = 96 ms^11^. To build the Glu‐only and GSH‐only phantoms, a hollow polyethylene sphere with a diameter of 10 cm was filled with saline (Milli‐Q water; 10% phosphate‐buffered saline) and 0.02% sodium azide to prevent bacterial growth over time, 3‐(trimethylsilyl)propionic (TSP) [1 mM] as the absolute reference at 0.00 ppm, and L‐glutamic acid [20 mM] or reduced L‐GSH [4 mM].

Next, we used a brain‐mimicking phantom containing Glu, GSH, GABA, NAA, Cr, Cho, and Gln where details of this phantom are outlined in our previous study (Kanagasabai et al. 2026) [7]. The brain‐mimicking phantom was built to (1) mimic the complexity of a human brain spectrum; (2) demonstrate selectivity and suppression; (3) aid in protocol optimization of double DANTE‐PRESS to refocus Glu and GSH simultaneously; (4) validate simulated fitting templates of Glu, GSH, and their co‐refocused metabolites; (5) perform a reproducibility assessment of the technique and protocol for in vivo tests; and (6) finally determine metabolite quantification precision.

### 2.2 Human Participants

Next, an in vivo proof‐of‐concept was performed in five healthy volunteers (two males and three females, ages 26–29 years). An MP2‐RAGE anatomical image (37 slices, TR/TE = 8000 ms/70 ms, flip angle = 120°, thickness = 3.5 mm, and field of view = 240 × 191 mm) was used as a reference to prescribe a 20 mm × 20 mm × 20 mm single ^1^H‐MRS voxel in the dorsal anterior cingulate cortex [11] [12]. Voxel placement was based on placing the posterior end of the voxel with the precentral sulcus and the caudal face of the voxel with the superior edge of the corpus callosum, and the voxel angle was set to be tangential to the surface of the corpus callosum.

For T_1_‐weighted anatomical images, we used the vendor‐provided MP2RAGE sequence (works‐in‐progress package 925B), the details of which are outlined in our previous work (Kanagasabai et al., 2026) [8, 12, 13]. This was used for white matter, grey matter, and cerebral spinal fluid tissue content estimation within the correction of the water concentration reference.

### 2.3 Magnetic Resonance Spectroscopy (MRS) Acquisition

First, a water‐suppressed PRESS (DANTE OFF) was acquired. Next, a water‐unsuppressed PRESS (DANTE OFF) was acquired. Then, a water‐selective DANTE pulse was centered on water to acquire a water‐unsuppressed DANTE‐PRESS spectrum. Finally, the Double DANTE‐PRESS pulse was centered on Glu's 2.35 ppm multiplet and GSH's 3.77 ppm singlet. Metabolite positions were determined relative to the NAA CH_3_ singlet (set to 2.01 ppm) observed in our water‐suppressed PRESS sequence and used for fine power and chemical shift adjustment loops of the DANTE pulse. The acquisition parameters of all sequences are outlined in Table 1.

**TABLE 1:** Acquisition parameters of Point RESolved Spectroscopy (PRESS) and double Delays Alternating with Nutations for Tailored Excitation Point RESolved Spectroscopy (DANTE‐PRESS).

Acquisition parameters	PRESS water unsuppressed	PRESS water‐suppressed	DANTE‐PRESS water unsuppressed	Double DANTE‐PRESS water suppressed
Voxel size	20 × 20 × 20 mm^3^ Phantom: isocenter In vivo: dACC			
TR	3000 ms			
TE	96 ms			
Phase cycle	8‐step			
Acquisition BW	3000 Hz			
Vector size	1024			
Number averages	8 + 4 dummy scans	64 + 4 dummy scans	8 + 4 dummy scans	64 + 4 dummy scans
WET water suppression	Only RF OFF	200 Hz	Only RF OFF (for DANTE‐correction)	200 Hz
DANTE FWHM	DANTE OFF	DANTE OFF	56 Hz = 0.19 ppm	
DANTE frequency domain period			2000 Hz	
Amplitude modulation			Gaussian (5% amplitude cut off)	
DANTE waveform			8192	
DANTE frequency‐selective pulse			Water at 4.7 ppm	Glutamate at 2.35 ppm and glutathione at 3.77 ppm and GABA at 1.89 ppm

### 2.4 Phantom Measurements

For all phantom experiments, a 2.0 × 2.0 × 2.0 cm^3^ voxel was prescribed at isocenter. Water‐unsuppressed and water‐suppressed spectra were acquired using the PRESS sequence with TR = 3000 ms, TE = 96 ms, NA = 8 for water‐unsuppressed, and NA = 64 for water‐suppressed, bandwidth = 3000 Hz, four dummy scans, 2048 sampling points, and phase cycling = 8‐step. The water‐unsuppressed signal was used as the reference peak for eddy current corrections (lineshape deconvolution reference) and performs absolute quantification (concentration reference). The water‐suppressed spectrum was acquired to identify in vivo references such as NAA to calculate the relative chemical shift of metabolites of interest. Water suppression was achieved by using the water excitation transfer (WET) module where it consists of four fixed variable flip angle Gaussian‐modulated RF pulses each surrounded by spoiler gradients [13].

Similarly, a water‐unsuppressed and water‐suppressed double DANTE‐PRESS spectra were acquired with the DANTE passband FWHM set to 56 Hz, the DANTE pulse waveform consisted of 8192 points, the DANTE frequency domain period was set to 2000 Hz (1/Δ*t*), and the Sinc FWHM (1/*τ*) set to 14,000 Hz. The DANTE chemical shift was first set to that of water for the water‐unsuppressed DANTE‐PRESS to quantify any signal loss due to the DANTE pulse ON condition compared with the standard PRESS water‐unsuppressed water signal. Next, the double DANTE‐PRESS chemical shifts were set to the gamma multiplet of Glu (at 2.35 ppm) and the cysteine singlet of GSH (at 3.77 ppm).

Metabolite centre frequencies were determined by running the DANTE chemical shift fine calibration loop with 0.01 ppm precision, implemented in the scanner vendor's image calculation extension (ICE), and centered on the NAA CH_3_ singlet (Appendix 1). The fine tune calibrations were specifically used to account for minor frequency and motion drifts where we did not observe drifts of 10–20 Hz due to physiological breathing as we are using a head‐only 7 T MR scanner. The frequency of the NAA CH_3_ singlet was assigned a chemical shift of 2.01 ppm. We then computed the frequency of Glu based on the known chemical shift difference between the NAA singlet (2.01 ppm) and Glu (2.35 ppm). The frequency of GSH (3.77 ppm) was determined by calculating the difference between it and Glu (2.35 ppm). The DANTE pulse center frequency could then be easily set to Glu and GSH simultaneously. This is where the amount of linear phase progression required for the Double‐DANTE pulse is determined. The pulse is generated online by the code of the pulse sequence in response to the user's input for desired frequencies.

To assess within‐session variability, we repeated the acquisition of Glu and GSH simultaneously 10 times using the same parameters. The chemical shift calibration loop on NAA was run every three acquisitions in order to compensate for any potential frequency drifts of the reference. To assess between‐session variability, a retest was performed at least a day apart from the original test.

### 2.5 In Vivo Measurements

Acquisition parameters similar to the phantom experiments were used for in vivo measurements. We obtained 10 measurements of Glu and GSH simultaneously to assess within‐session variability. Healthy volunteers were scanned twice; at baseline to assess the within‐session variability and at least 1 week later to assess between‐session variability. This study was approved by the Health Science Research Ethics Board (HSREB), file number 120093, of the University of Western Ontario. Table 1 provides a detailed breakdown of the acquisition parameters used in this study.

### 2.6 Spectral Quantification

**FIGURE 4:**
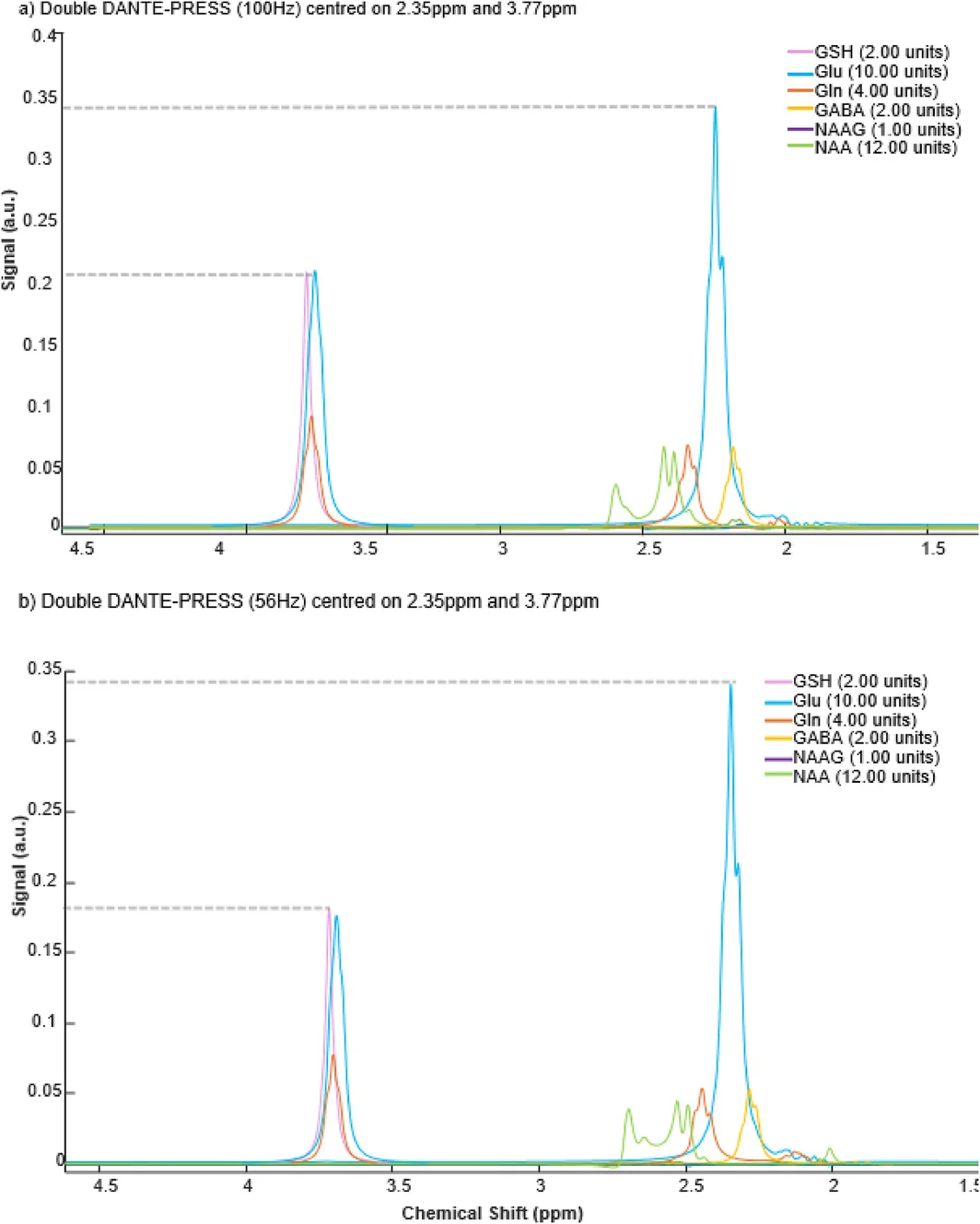
FID‐A simulation reflects brain‐representative concentrations of glutamate (10 mM), glutamine (4 mM), glutathione (2 mM), GABA (2 mM), NAAG (1 mM), and NAA (12 mM) at 2.35 and 3.77 ppm using double DANTE‐PRESS passbands of (a) 100 Hz and (b) 56 Hz with all other acquisition parameters the same as reported in Table 1.

FID‐A was used to generate simulated spectral fitting templates of double DANTE‐PRESS with a DANTE passband of 56 Hz centered at 3.77 and 2.35 ppm in vivo (Figure 4). Furthermore, we show the effect of metabolite signal relative to DANTE passband size to show the reduced effect on co‐refocused metabolites (Figure 4). Spectra visualization and metabolite fitting were done using an in‐house post‐processing software known as FitMan [14]. FitMan is a time‐domain fitting algorithm that uses a nonlinear, iterative Levenberg–Marquardt minimization technique to estimate metabolite chemical shift, concentration, linewidth, and phase (zeroth order and first order). Double DANTE‐PRESS simplifies spectral signatures by refocusing two metabolites of interest while suppressing unwanted signals using the crusher gradients. Coefficients of variance (% CV) and Cramer lower bounds (CRLBs) were calculated to evaluate the intraindividual and inter‐individual (group) variability, lowest observable amount of uncertainty, and reproducibility of the DANTE‐PRESS sequence. Given the long echo time, there were no macromolecule contaminations. No T_1_‐ and T_2_‐weighting corrections were implemented [14, 16]. Partial volume corrections were done using FSL‐FAST [16].

## 3 Results

FID‐A simulations of the double DANTE‐PRESS centered on 2.35 and 3.77 ppm using a passband of 100 and 56 Hz demonstrated a reduction in contributions from co‐refocused metabolites while preserving the dominant Glu multiplet at 2.35 ppm and GSH‐weighted signal at 3.77 ppm using a narrower passband (Figure 4a,b).

**FIGURE 5:**
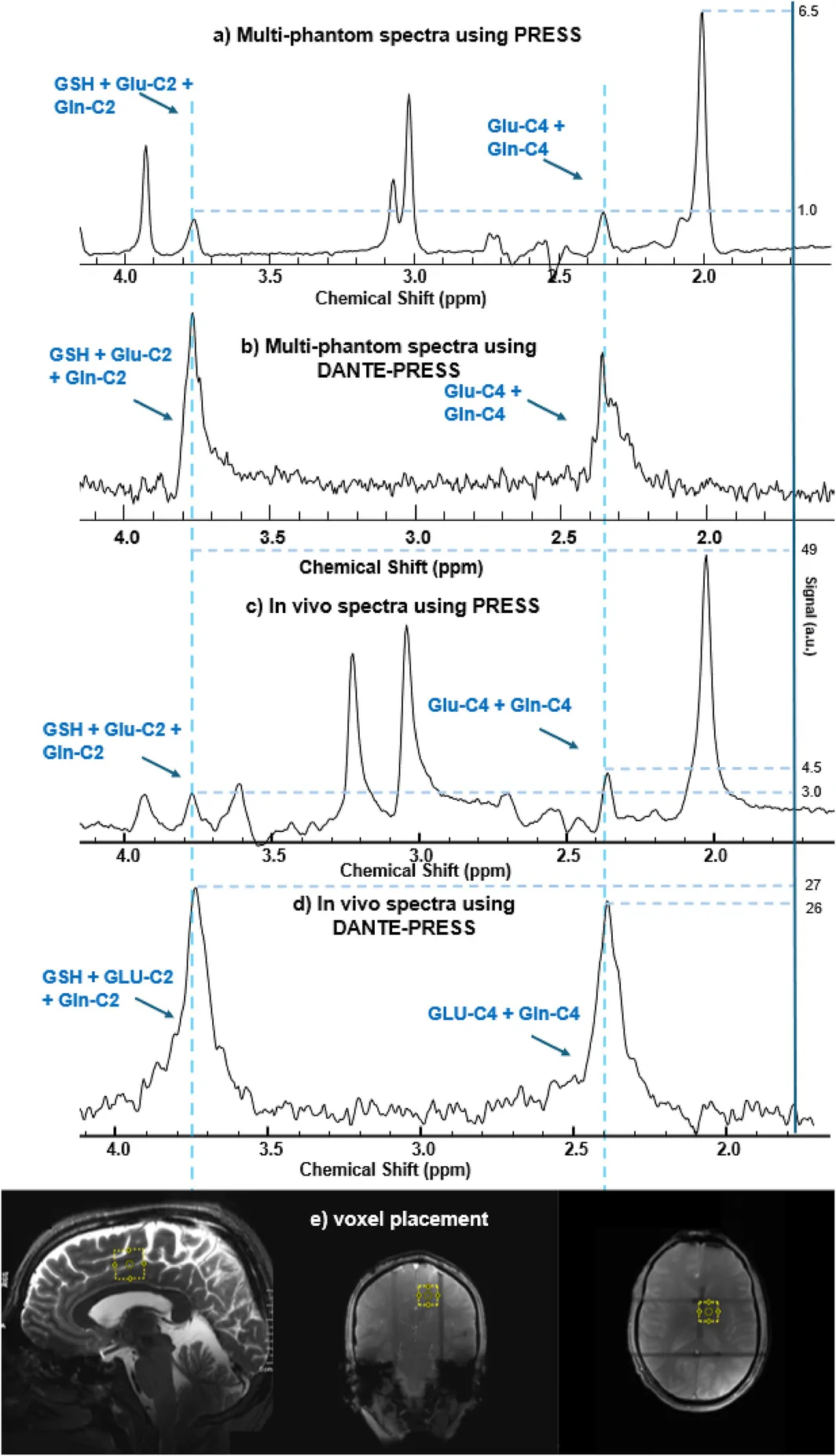
Water suppressed spectra (TR/TE/AVG = 3000 ms/96 ms/64avg) from a brain‐mimicking phantom contain Glu, Gln, GSH, GABA, NAA, Cr, and Cho using (a) Point RESolved Spectroscopy (PRESS) and (b) Delays Alternating with Nutations for Tailored Excitation Point RESolved Spectroscopy with the DANTE pulse (56 Hz) centered on Glu at 2.35 ppm and GSH at 3.77 ppm (DANTE passband = 56 Hz) simultaneously using the 7 T and in a healthy volunteer using (c) PRESS (d) DANTE‐PRESS using the same acquisition parameters as the phantoms and (e) two‐dimensional sagittal anatomical image was used as a reference to prescribe a 2.0 × 2.0 × 2.0 cm^3^ single 1H‐MRS voxel in the anterior cingulate cortex of a healthy volunteer. All panels have labelled signal amplitudes (a.u.) for Glu and GSH.

Using a brain‐mimicking phantom (containing NAA, Glu, Gln, GABA, GSH, Cr, TSP, and 0.025% Azide), double DANTE‐PRESS was able to simplify and selectively refocus metabolites within its passband at 2.35 and 3.77 pm compared with the conventional symmetric PRESS (Figure 5a,b).

In phantoms, the mean %CV of Glu and GSH concentration estimates were less than 5% and 7%, respectively, and the mean %CRLB of Glu and GSH were less than 2% and 3%, respectively within Sessions 1 and 2 (Table 2).

**TABLE 2:** Mean % Cramer Rao Lower bounds (CRLB), mean % coefficient of variation (CV), and mean concentration of glutamate at 2.35 ppm and glutathione at 3.77 ppm using double DANTE‐PRESS in (a) phantoms and (b) in vivo at 7 T. Each value is the mean of 10 measurements in phantoms or in vivo within single session.

(a)							
Metabolite of interest in phantoms		%CV Session 1 (*n* = 10)	%CRLB Session 1 (*n* = 10)	%CV Session 2 (*n* = 10)		%CRLB Session 2 (*n* = 10)	
Glu		4.71	0.21	4.37		1.37	
GSH		5.69	2.78	6.45		0.79	
(b)							
Metabolite of interest in healthy volunteers		Intraindividual variability					
		%CV Session 1 (*n* = 10)	% CRLB Session 1 (*n* = 10)	Concentration Session 1 (mM)	%CV Session 1 (*n* = 10)	% CRLB Session 2 (*n* = 10)	Concentration Session 2 (mM)
Glu	1	4.97 ± 0.023	3.85 ± 0.007	10.38 ± 0.0004	3.81 ± 0.015	3.62 ± 0.006	9.12 ± 0.0003
	2	6.34 ± 0.021	5.27 ± 0.007	8.39 ± 0.0005	5.41 ± 0.018	3.85 ± 0.007	8.75 ± 0.0009
	3	5.69 ± 0.021	4.68 ± 0.007	7.78 ± 0.0004	5.55 ± 0.023	3.75 ± 0.006	8.30 ± 0.0008
	4	7.89 ± 0.020	5.91 ± 0.010	8.93 ± 0.0009	6.29 ± 0.025	5.78 ± 0.008	9.89 ± 0.0006
	5	4.77 ± 1.70	4.73 ± 0.84	8.41 ± 0.0004	11.07 ± 0.36	5.88 ± 0.75	7.89 ± 0.0008
	Inter‐individual variability (%) (*n* = 5)	5.93 ± 1.26	4.89 ± 0.76	8.78 ± 0.98	6.43 ± 2.75	4.58 ± 1.15	8.79 ± 0.77
GSH	1	9.51 ± 0.016	7.26 ± 0.017	2.53 ± 0.0002	9.38 ± 0.123	9.09 ± 0.007	1.98 ± 0.0002
	2	9.15 ± 0.013	8.86 ± 0.009	1.21 ± 0.0002	8.54 ± 0.012	7.25 ± 0.013	1.18 ± 0.0001
	3	10.25 ± 0.016	8.65 ± 0.021	2.14 ± 0.0002	8.34 ± 0.010	10.26 ± 0.014	1.66 ± 0.0001
	4	9.36 ± 0.008	8.44 ± 0.026	2.97 ± 0.0001	9.87 ± 0.018	8.68 ± 0.011	3.01 ± 0.0003
	5	10.10 ± 1.51	9.84 ± 0.28	2.29 ± 0.0002	12.27 ± 0.97	12.14 ± 0.87	2.45 ± 0.0001
	Inter‐individual variability (%) (*n* = 5)	9.67 ± 0.48	8.30 ± 0.716	2.22 ± 0.65	9.68 ± 1.57	9.48 ± 1.83	2.06 ± 0.71

**FIGURE 6:**
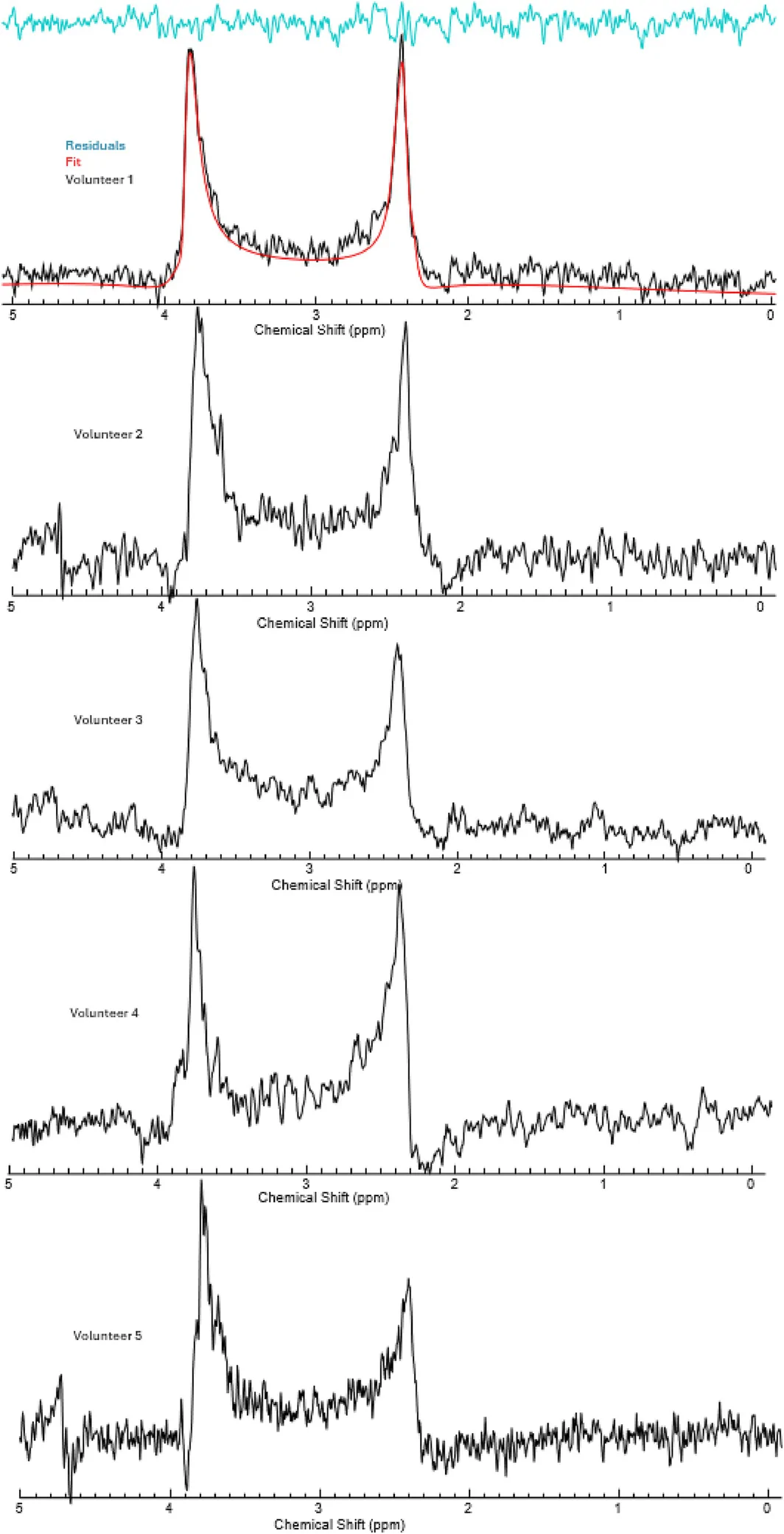
Full in vivo brain spectra from all five healthy volunteers using double DANTE‐PRESS centered on 2.35 and 3.77 ppm. Raw data with water removed and 2 Hz exponential filter applied for visualization purposes. Representative spectral fit and residuals shown for Volunteer 1.

Following simulation and phantom validation, double DANTE‐PRESS was evaluated in five healthy volunteers (Figures 5c,d, and 6). Double DANTE‐PRESS demonstrated that the narrowband frequency‐selective pulses produced simplified in vivo spectra containing dominant signals from Glu at 2.35 ppm and GSH‐weighted signal at 3.77 ppm simultaneously.

Measurements of Glu and GSH in five healthy volunteers were averaged to get the inter‐individual mean within two sessions (baseline and retest) %CV and %CRLB. In vivo, the inter‐individual mean %CV of Glu and GSH were less than 7% and 10%, respectively, and %CRLB were less than 5% and 10%, respectively, across two sessions (Table 2).

## 4 Discussion

This is the first in vivo study that reported measurements of Glu at 2.35 ppm and GSH at 3.77 ppm simultaneously using double DANTE‐PRESS at 7 T with %CVs and %CRLBs less than 7% for Glu and 10% for GSH across sessions (Figure 5).

Double DANTE‐PRESS is a single‐shot multifrequency‐selective sequence that selectively refocuses signals from two passbands simultaneously while suppressing unwanted coherences. In this study, the DANTE‐PRESS frequency‐selective passband was 56 Hz wide, which is equivalent to 0.19 ppm (Figures 5 and 6). The narrow DANTE passbands (two customizable frequency‐selective pulses) allowed for a simpler spectral modeling template without using spectral editing (Figure 4). Unlike spectral editing techniques, double DANTE‐PRESS does not rely on subtraction of interleaved acquisitions. This avoids subtraction artifacts, reduces sensitivity to subject motion and frequency drift, and improves SNR efficiency per unit time [5]. However, editing techniques may offer improved spectral specificity for coupled spin systems and are the current standard for GSH detection [17].

To our knowledge, this is the first study of double DANTE‐PRESS reporting on measurements from GSH at 3.77 ppm and Glu at 2.35 ppm. Double DANTE‐PRESS is intended as a complementary frequency‐selective acquisition strategy alongside STEAM, PRESS, sLASER, and spectral‐editing approaches. DANTE‐PRESS provides an alternative balance between frequency‐selective refocusing, simplified spectral modelling, acquisition efficiency, and simultaneous targeting of multiple spectral regions without subtraction editing. Most clinician‐initiated investigations target one or two biomarkers, which explains why most sequences are often optimized to report one or two metabolites at a time. As the precision for one metabolite is improved, the precision of the secondary metabolite is reduced [6]. Furthermore, double DANTE‐PRESS can be optimized to target 2–4 metabolites at a time by tailoring sequence parameters such as the frequency domain period and the frequency of the next repeating passband, to the metabolites of interest, or by increasing the bandwidth of the DANTE passband to include more metabolites of interest [7]. Despite producing simplified brain spectra, the work presented here is part of the early iterations of double DANTE‐PRESS development and it is very likely that the acquisition parameters of the sequence could be further optimized to produce more metabolite‐selective spectra.

DANTE‐PRESS is not meant to replace non‐frequency‐selective or spectral editing approaches but rather complement these techniques. DANTE‐PRESS acquisitions could be paired with an optimized‐PRESS acquisition to provide end users with the full brain spectra along with their frequency‐selective spectra. Although DANTE‐PRESS does not achieve selectivity through J‐coupling networks achieved in spectral editing, its single‐shot acquisition, reduced scan times, reduced subtraction‐related motion artifacts, and lower SNR challenges from subtraction‐based editing may be advantageous for clinical populations with lower tolerance to protracted scan times and difficulty remaining immobile.

### 4.1 Repetition Time, Echo Time and DANTE Passband

In our previous work, we introduced single DANTE‐PRESS where we used a DANTE passband of 100 Hz (TE = 68 ms). We optimized the DANTE centre frequency to refocus metabolites from the 2.35 ppm (aiming at Glu ^4^C) and 2.01 ppm (NAA singlet) spectral regions independently. One of the limitations of our previous work was using a relatively large passband (100 Hz) where neighboring metabolites within the passband were co‐refocused [7]. However, by capitalizing on the increased spectral dispersion observed at 7 T and working with relatively large concentration metabolites such as NAA and Glu, the achieved %CRLB and %CV were still < 5% for both metabolites.

In this study, we used a DANTE passband of 56 Hz (TE = 96 ms). This passband was approximately half the passband of our previous work; however, we still observed co‐refocused metabolites specifically at the 3.77 ppm passband. The GSH singlet is at 3.77 ppm, Glu alpha multiplet is at 3.74 ppm, and the Gln alpha multiplet is at 3.75 ppm. Although metabolite covariance matrices were not computed, the overlap between GSH, Glu, and Gln at 3.77 ppm was evaluated qualitatively using the FID‐A simulation of the DANTE pulse at 3.77 ppm (Figure 4). This demonstrated that narrowing the DANTE passband from 100 to 56 Hz reduces contributions from the co‐refocused Glu ^2^C and Gln ^2^C while retaining a GSH‐weighted resonance at 3.77 ppm.

To reduce contributions from neighboring metabolites, the DANTE pulse could be even narrower; however, this comes with a long TE trade‐off as the passband width increases the DANTE pulse duration and increases the minimum TE. This could be advantageous as it could further dampen contributions from co‐refocused metabolites as we can see in our simulation between a wider versus narrow DANTE passband but also eliminate metabolites with shorter T_2_.

Alternatively, Glu ^2^C contributions to GSH singlet could be modelled using Glu ^4^C, assuming T2 values are the same. However, with the current protocol setup, the main source of error in measuring GSH at 3.77 ppm comes from the overlapping Gln ^2^C. A potential solution could be to acquire an additional spectra where the center of the DANTE passband is on Gln ^4^C and the Gln ^2^C contributions to GSH at 3.77 pm could be modelled based on Gln ^4^C.

### 4.2 T1/T2 Corrections

With a long TE (96 ms) and short TR (3000 ms), this could introduce T_1_‐ and T_2_‐weighted contributions. We did not perform T_1_ or T_2_ corrections in this study because the primary objective of this study was to introduce and validate our novel pulse sequence and demonstrate its feasibility in vivo.

Relaxation corrections are a crucial step when interpreting metabolite concentrations and a limitation of our study as we did not collect T_2_ measurements of each participant. A J‐suppression PRESS study at 7 T reported on T_2_ values of Glu to be 184 ± 18 ms within the frontal grey matter region [18]. Based on these published measurements, approximately 60% of the Glu signal is retained at a TE = 96 ms. Furthermore, Wong et al. recommended TE ranges from 80 to 105 ms may be optimal when targeting Glu due to complex J‐coupling and its variability in Glu measurement with shorter TEs [20, 21]. A MEGA‐sLASER study at 7 T reported on T_2_ values of GSH to be 145.0 ± 20.1 ms within the occipital lobe [21]. Based on these values, approximately 51% of the GSH signal is retained at TE = 96 ms.

Although literature values could provide direction for T_1_/T_2_ correction, relaxation times are sequence‐dependent and influenced by J‐coupling evolution. Applying literature‐based corrections may introduce additional bias, and these corrections do not influence spectral selectivity or detectability achieved by double DANTE‐PRESS. Nonetheless, the reported metabolite concentrations should be interpreted as T_1_/T_2_‐weighted estimates rather than absolute concentrations. Future studies involving larger sample size should incorporate metabolite‐specific relaxation corrections or direct measurements to improve accuracy.

### 4.3 Chemical Shift Displacement Error (CDSE)

CDSE remains a known limitation of PRESS localization specifically at ultrahigh fields [22]. CSDE arises from slice‐selective excitation and refocusing pulses. The DANTE pulse is a frequency‐selective pulse and therefore does not add to CDSE effects. Sequences such as sLASER are known to reduce the effect of CDSE and could be a promising sequence for the implementation of the DANTE pulse [20, 24]. The tradeoff would be that it would require a longer minimum TE and TR to accommodate for all the pulses in addition to achieving a narrowband DANTE pulse.

Despite CSDE and T2 effects, we opted to implement double DANTE‐PRESS at 7 T due to its increased spectral dispersion and SNR, which provided an advantageous development platform to introduce a novel technique. However, DANTE‐PRESS may be beneficial at lower field strengths (1.5 or 3 T) where spectral overlap is more pronounced, benefits from spectral simplicity, and is also clinically feasible [8].

### 4.4 Frequency Drift and Motion Artifacts

The DANTE pulse is sensitive to scanner frequency drifts and participant motion when averaging multiple transients. This could result in signal loss. A narrow DANTE passband is advantageous in creating simple in vivo spectra and improved detection of low concentration metabolites near abundant metabolites. However, the narrower the frequency profile of the DANTE pulse, the longer the DANTE pulse duration in the time‐domain. This could become a concern when the DANTE passband becomes comparable with size of the change in scanner drift (i.e., in our case, DANTE passband < 20 Hz). Using a DANTE passband of 56 Hz was a reasonable threshold where it was not affected by frequency drifts but narrow enough to create a simpler spectrum. We performed manual adjustments to the centre frequency after each acquisition to ensure drifts would remain under 5 Hz over the duration of the acquisition (3 min 14 s). Furthermore, we performed fine calibrations (step size 0.01 ppm ~ 3 Hz) of the DANTE pulse frequency on the NAA singlet at 2.01 ppm prior to each DANTE‐PRESS acquisition. This was to account for any drift that may occur and affect the frequency‐selectivity of the DANTE pulse. Depending on the user and the length of acquisition, this may increase the overall scan time by 1–3 min; however, this fine calibration step enables acquisition of high‐quality spectra.

Participant motion artifacts are also a common concern in spectroscopy and could affect frequency selectivity when using a narrow DANTE pulse passband. This is a concern when working with long scan times with high number of averages to improve detection of low concentration metabolites. An advantage of DANTE‐PRESS is not having to acquire additional spectra to perform spectral editing (i.e., addition or subtraction techniques). In this study, the scan time for our study was 3 min and 14 s for 64 averages. Other frequency/metabolite‐selective techniques such as MEGA‐sLASER and MEGA‐PRESS require approximately double the scan time for the same number of spectral points [25, 26, 27]. To minimize participant motion, we placed pads within the head‐coil while ensuring comfort. Participants were requested to remain still during acquisitions; hence, all acquisitions from our study were usable. However, if there is significant head motion, it will suffer from the motion artifacts like other MRS sequences.

### 4.5 Macromolecule Contamination

Studies that have used metabolite‐selective techniques such as MEGA‐sLASER or MEGA‐PRESS have expressed concerns on macromolecular contamination [28, 29]. Macromolecules are broad signals prominent around 0.5–2.0 ppm, 2.08 ppm, 2.25 ppm, and 3.00 ppm where they tend to be co‐edited with GABA multiplets at shorter echo times. At 7 T, it was recommended to use a TE > 100 ms to minimize the effect of macromolecule contributions. In our study, with a DANTE passband of 56 Hz, the minimum TE was 84 ms; however, we have used a TE = 96 ms to nearly meet this recommendation and minimize the effect of macromolecules (Figure 6). Furthermore, according to the consensus table of main macromolecule peaks, they did not report on values at our chemical shifts of interest (2.35 and 3.77 ppm) [29]. There are other factors to account for that could affect macromolecule contributions such as brain regions and inter‐individual variations on macromolecule contributions (GM, WM, and CSF contributions); however, there are still ongoing investigations of tissue type and macromolecule contributions [29]. Furthermore, Wijtenburg commented on the role of metabolite fitting errors when using basis sets that contain inaccurate representations of macromolecules as they can negatively impact the fitting accuracy and quantification [30]. However, this is a limitation of the study as we did not experiment with macromolecule phantoms or simulations.

### 4.6 Spectral Complexity and Modelling

Quantification precision depends on the raw spectral quality (data variability) and complexity of the quantification template (algorithmic variability). When working with frequency‐selective techniques, it is important to account for metabolites that are present in the DANTE passband in vivo; otherwise, this will introduce fitting errors (i.e., underestimate or overestimate concentrations) [30]. We report on CRLBs to measure the variability of a spectral fit parameter and group CVs to measure the total group variability [6]. Both these metrics can vary based on the number of metabolites in a spectrum, which influences the number of modelling parameters required for a fitting algorithm thereby driving algorithmic variability. The total variability encompasses algorithmic variability (reported as CRLB) along with inter‐individual variability (CV). Several MRS studies tend to optimize for one metabolite and report on additional metabolites if they are within the CRLB threshold. However, modelling a non‐frequency‐selective technique (for the same number of FID data points as a frequency‐selective technique) just to report on one or two metabolites requires complex basis sets to model the broad spectra and increases modelling variability [31].

The double DANTE‐PRESS has two narrowband frequency‐selective passbands where its spectra can be quantified using a simpler model with less degrees of freedom reduce algorithmic variability. Because this is the first study of double DANTE‐PRESS centered on Glu at 2.35 ppm and GSH at 3.77 ppm, there is no prior literature describing the response of spin systems for this pulse, so we opted to build fitting templates from data acquired in single‐metabolite phantoms to develop prior knowledge. Because the Glu C2 doublet of doublet is only 0.03 ppm away from the GSH singlet, the DANTE pulse refocused some of the Glu C2 signal as well. To account for this spectral contamination, we tested our acquisition protocol on a Glu and Gln phantom to assess and model the Glu signal that was refocused using the acquisition parameters used to target GSH. Using phantoms, we were able to determine and model the relationship between parameters for the individual metabolites before moving to multi‐metabolite phantoms and in vivo. Alternatively, Glu signal could be selectively removed by using an asymmetric echo time [33, 34]. We moved on to simulations of the double DANTE‐PRESS pulse sequence using the acquisition parameters from our protocol to generate custom fitting templates (i.e., basis sets) for FitMan using the FID‐A toolbox [34]. The next step of this study would involve a larger sample of healthy participants as fodder for power calculations of a study of human brain.

Although we acquired a water‐suppressed symmetric PRESS spectra, it was not included in a quantitative comparison with DANTE‐PRESS. The acquisition (64 avg) was primarily used for identifying the in vivo chemical shift reference (NAA) for fine‐tune calibrations of the DANTE pulse. Due to time constraints, repeated acquisitions of PRESS (10 within‐session acquisitions) to assess variability in precision and accuracy were not feasible. This would have required an additional 45 min of scan time for an in vivo proof‐of‐concept study. Moreover, the acquisition parameters of this study were optimized for double DANTE‐PRESS, thereby creating a negative bias towards PRESS.

Nevertheless, the water‐suppressed PRESS spectra (Figure 5a,c) can provide a qualitative comparison with double DANTE‐PRESS (Figure 5b,d). Visually, the double DANTE‐PRESS spectra create a simple spectrum for simple spectral modelling. The reduced spectral complexity thereby has downstream effect reducing algorithmic variability upon fitting and quantification relative to the symmetric PRESS spectra. Future work should include a comparison of ultrashort/short/TE‐optimized PRESS, STEAM, and/or semi‐LASER, and DANTE‐PRESS to provide a critical quantitative comparison among the four sequences using their respective optimized acquisition parameters.

### 4.7 Test–Retest Studies Using Single Shot Sequences

We used a TE = 96 ms, which is consistent with other PRESS studies who have completed test–retest evaluations in the ACC where many of these studies have reported GSH measurements at the 2.54 ppm multiplet. Furthermore, these studies were often optimized for one metabolite and report secondary metabolites if the %CRLB and %CV are lower than a given threshold.

Lally et al. used a TE‐optimized PRESS (TE1 = 69 ms and TE2 = 37 ms, TR = 2500 ms, and AVG = 128) along with a J‐suppression pulse to reduce NAA multiplet signals at 2.49 ppm at the pregnual ACC [35]. They reported %CRLBs < 2% and < 7.5% for Glu and GSH, respectively. However, they also reported %CV < 8% and < 15% for Glu/Cr and GSH/Cr, respectively, at 7 T. This study used 128 averages, which increased the scan time and may have increased participant motion and scanner drift. It was observed with shorter acquisition times, less than 5 min; it was observed to improve %CRLBs and %CVs. However, with a short echo time, it makes it challenging to resolve low concentration metabolites from larger metabolites. Furthermore, there could be contributions from macromolecules at shorter TEs. For example, Witjtenburg et al. used a short‐TE STEAM (TE = 14 ms, TR = 3000 ms, TM = 25 ms) where the %CRLB were < 2% and < 6.7% and the %CV were < 8% and < 16% for Glu and GSH, respectively [30]. Prinsen et al. used J‐difference editing sLASER (TE = 72 ms, TR = 3000 ms and AVG = 128) to report measurements of GSH with %CRLB < 7% and %CV < 11% [36]. Using STEAM (TE = 10 ms, TM = 50 ms TR = 3000 ms, AVG = 96), they reported measurements of Glu with %CRLBs < 7% and %CV < 5%. Using two different sequences to optimize for one or two metabolites increases scan times including the additional calibration steps required for precise measurements.

The GSH singlet at 3.77 ppm often has strong overlap with the Glu multiplet at 3.74 ppm and Gln multiplet at 3.75 ppm. As such, the measurements of GSH at 3.77 ppm are best described as “GSH‐weighted” and are heavily dependent on the quality of the model basis sets. Most studies report on the GSH multiplet at 2.54 ppm due to improved spectral specificity; however, spectral editing is required (i.e., MEGA‐sLASER and MEGA‐PRESS) [38, 39]. The trade‐offs associated with spectral editing techniques include lower SNR, more prone to frequency drift or motion errors, higher SAR level, limited TE optimization, and longer acquisition times required for optimal calibration. Furthermore, there could be subtraction artifacts where co‐edited signals are refocused whereby reducing quantification accuracy. With our approach, we centered the DANTE pulse on Glu at 2.35 ppm and GSH singlet at 3.77 ppm simultaneously. We chose to refocus the GSH singlet at 3.77 pm because it is an attractive spot for a short TE single shot sequence such as short TE PRESS or sLASER especially at ultrahigh field strengths [40, 41]. The DANTE pulse is a frequency‐selective technique where the narrowband passband selectively refocuses signal within its passband and suppresses signal outside of it. We used an echo time of 96 ms to achieve a DANTE passband of 56 Hz, which is long for a traditional PRESS, but it provides SNR equivalent to a short‐TE PRESS.

### 4.8 Clinical Applications

Simultaneous detection of Glu and GSH is particularly relevant for investigations of redox balance and excitatory neurotransmission in neurological and oncological disorders. In oncology, elevated Glu levels could drive tumor proliferation and metabolism whereas GSH plays a central role in radiation and/or chemotherapy resistance as a response to oxidative stress [42, 43]. Glu and GSH could therefore be potential therapeutic targets for longitudinal monitoring of treatment response where they can be detected and quantified by double DANTE‐PRESS, a single‐shot, multifrequency‐selective technique.

DANTE‐PRESS could be applied to other organs to assess biomarkers of breast or prostate cancer. Most studies in oncology monitor the abundant singlets such as lactate, choline, and citrate partly due to spatial and temporal limitations using phosphorus or proton MRS and the ability to selectively detect low in vivo concentration metabolites [43]. There is a gap to selectively assess low in vivo concentration metabolites such as Glu and GSH to understand the tumor microenvironment in response to treatments, specifically monitoring the effects of radiation‐induced oxidative stress and their recovery [45, 46]. Furthermore, this could provide insight on personalized medicine based on their metabolic fingerprint and be used in a longitudinal or preclinical drug development study [46].

## 5 Conclusion

Double DANTE‐PRESS (TE = 96 ms) is a novel technique that produces simplified spectra enriched in Glu‐C4 and GSH‐weighted signals at 2.35 and 3.77 ppm, respectively, while substantially suppressing signals outside these passbands in a multi‐metabolite phantom and in vivo at 7 T. The approach allowed for the inter‐individual mean % CV and %CRLB to be both < 10% in vivo without the use of spectral editing and features single‐shot localization and single‐shot metabolite selectivity. These features provide improved temporal resolution compared with multi‐shot techniques, improved resilience to motion via frequency‐phase‐coherent averaging, and spectral signatures containing 1 or 2 metabolites allowing for simple spectral modelling, thereby reducing algorithmic variability.

## Author Contributions

Kanagasabai was responsible for study conception and design, acquisition of data, analysis and interpretation of data, drafting of manuscript, and critical revision. Oran was responsible for acquisition of data, drafting of manuscript, and critical revision. Palaniyappan was responsible for drafting of manuscript and critical revision. Theberge was responsible for study conception and design, acquisition of data, analysis and interpretation of data, drafting of manuscript, and critical revision.

## Funding

Partial stipend support of KK and support of scanner user fees by NSERC Discovery Grant (RGPIN‐2022‐04425) to JT and CIHR Foundation Grant (FDN 154296) to LP and Canada First Excellence Research Fund to BrainSCAN, Western University (Imaging Core); LP's research is supported by the Canada First Research Excellence Fund, awarded to the Healthy Brains, Healthy Lives initiative at McGill University (through a New Investigator Supplement) and Monique H. Bourgeois Chair in Developmental Disorders. He receives a salary award from the Fonds de recherche du Québec‐Santé (FRQS); NSERC to KK.

## Data Availability

The data that support the findings of this study are available on request from the corresponding author. The data are not publicly available due to privacy or ethical restrictions.

## Appendix

Screen captures of scanner's real‐time Inline Display window for fine calibration of (a) excitation flip angle, (b) refocusing flip angle, and (c) DANTE pulse frequency. Calibration modes consisted of 10 measurements in a user‐specified degree/ppm increment. The spectra for all 10 measurements were plotted in the same window, and the spectrum of the optimum value was automatically indicated with a different color (magenta).
